# Li—Co Dual‐Doped Ceria‐Based Composite as a Promising Low‐Temperature Electrolyte for Metal‐Supported Solid Oxide Electrolyzers

**DOI:** 10.1002/cssc.202501679

**Published:** 2025-11-03

**Authors:** Yuheng Liu, Ming Xu, Wei Zhang, Yunlong Zhao, Bahman Amini Horri

**Affiliations:** ^1^ School of Chemistry and Chemical Engineering Faculty of Engineering & Physical Sciences University of Surrey Guildford Surrey GU2 7XH UK; ^2^ Advanced Technology Institute Faculty of Engineering & Physical Sciences University of Surrey Guildford Surrey GU2 7XH UK; ^3^ Dyson School of Design Engineering Faculty of Engineering Imperial College London London SW7 2AZ UK

**Keywords:** codoped solid‐state electrolytes, electrical conductivity, gadolinium‐doped ceria, low‐temperature sintering aid, metal‐supported solid oxide electrolysis cells

## Abstract

Solid oxide electrolysis cells (SOECs) are among the most efficient energy‐conversion devices for power‐to‐X applications in green energy technologies. Here, we report a high‐level (5 mol%) Li‐ and Co‐dual‐doped gadolinium‐doped ceria (GDC) electrolyte synthesized under an inert atmosphere, suitable for fabricating SOECs using conventional ferritic steel supports. The doped GDC exhibits uniform dopant incorporation and a single‐phase cubic fluorite structure, achieving 98.18% relative density at 950 °C. Dilatometry and microstructural analyses reveal that Li–Co codoping significantly reduces sintering temperature and improves grain connectivity. Time‐of‐flight secondary ion mass spectrometry shows a Li,Co‐rich surface layer whose thickness depends on sintering conditions, while Raman spectroscopy confirms the presence of a LiCoO_2_ phase and temperature‐dependent oxygen‐vacancy concentration. Electrochemical impedance spectroscopy demonstrates enhanced ionic conductivity, particularly for the sample sintered at 950 °C (denoted 5LC‐4), which achieves increases of 269.5% at 450 °C and 138.85% at 750 °C compared with commercial GDC. The ionic conductivity reaches 2.17 × 10^−2^ S cm^−1^ with an activation energy of 0.32 eV. A symmetric five‐layer SOEC integrating 5LC‐GDC exhibits superior electrochemical performance to yttria‐stabilized zirconia (YSZ) support, achieving a peak power density of 267.5 mW cm^−2^ at 850 °C.

## Graphical abstract

Enhanced sinterability and ionic conductivity are achieved with Li and Co dual‐doped GDC electrolytes for metal‐supported solid oxide electrolyzers. The optimized sample shows 2.17 × 10^2^ S cm^1^ at 750 °C, which typically approaches the superionic solid electrolyte conductivity ranges. A tubular cell fabrication and analysis demonstrated its potential as a high‐performance electrolyte for metal‐supported SOCs.

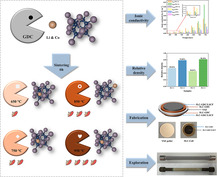

## 1 Introduction

Solid oxide electrolysis cell (SOEC) technology is esteemed as an ideal energy conversion apparatus for the electrolysis process in power‐to‐X solutions, owing to its unparalleled conversion efficiencies resulting from favorable thermodynamics and kinetics at higher operating temperatures (500–900 °C).^[^ ^1^ ^]^ Facilitated by renewable energy sources such as solar and wind energy, SOEC technology demonstrates the capacity to generate hydrogen and hydrocarbons through water electrolysis and carbon dioxide reduction and can even extend its capabilities to produce ammonia through nitrogen reduction.^[^ ^2^ ^]^

Conventional cermet‐based SOECs face considerable challenges for automobile applications and commercialization, which include slow start‐up and cool‐down rates, fragile cermet‐based cells and relatively high material costs.^[^ ^3^ ^]^ With the development of technology for SOECs, metal‐supported SOECs (MS‐SOECs) are considered as an advanced structure design over all the conventional cermet‐based SOECs due to their advantages, due to their versatility (e.g., rapid start‐up and cool‐down without cracking) and their lower material cost (e.g., the stainless steel support that is commonly used for MS‐SOECs is much cheaper than the Ni‐based cermet used for conventional anode‐supported cells).^[^ ^4^ ^]^

However, the sintering temperature in the fabrication process is one of the main challenges for MS‐SOECs. Gadolinium‐doped ceria (GDC) is the most commonly used solid electrolyte material for intermediate‐temperature (below 800 °C) SOECs because it exhibits high ionic conductivity at these temperatures.^[^ ^5^, ^6^, ^7^, ^8^ ^]^ The typical sintering temperature for GDC is higher than 1350 °C, which restricts its applications in metal‐supported cells. Compared with conventional ceramic support, the porous metal substrate cannot withstand high temperatures (>1000 °C) because it will oxidize and become unable to sustain a structural load.^[^ ^9^ ^]^ Also, the high‐temperature treatment could damage the structure and porosity of the metal support, weakening the mechanical strength.^[^ ^10^ ^]^

The reason for using the high sintering temperature for solid electrolytes is to ensure that the prepared electrolyte is gas‐tight. Therefore, obtaining solid electrolyte material with a relatively low sintering temperature is crucial. The sintering temperature of electrolyte material is decided by the grain‐boundary diffusion rate between particles during sintering, which diffusion can be described via the flux of atoms along a grain boundary.^[^ ^11^ ^]^

(1) J=MCΔμ

*J* is the flux of atoms; *M* is the atomic mobility along the grain boundary; *C* is the vacancy concentration; Δ*μ* is the driving force for sintering. Increasing any *M*, *C*, or Δ*μ* by adding doping elements will decrease the sintering temperature theoretically. For instance, cobalt doping for GDC could alter M and increase ∇*μ* due to capillary effects.^[^ ^12^ ^]^ In past decades, various attempts have been made to reduce the sintering temperature of the GDC materials by adding different doping elements.^[^ ^13^, ^14^, ^15^, ^16^, ^17^, ^18^, ^19^ ^]^ According to Nicholas's research, Cu, Co, Fe, Mn, Li, and Zn can reduce the sintering temperature of GDC.^[^ ^20^ ^]^ Chen's group reported that 5 mol% Li doping can get dense GDCs with obvious sintering temperature drops.^[^ ^21^ ^]^ These previous studies have shown that doping is an efficient method to reduce the sintering temperature of GDC.

However, the sintering temperature of GDC is still beyond 1000 °C in most of the research.^[^ ^6^, ^20^, ^21^, ^22^, ^23^, ^24^, ^25^, ^26^ ^]^ This sintering temperature will still affect the steel base support and increase the material and fabrication costs. Some studies have reported that doped GDC with a low sintering temperature (850–900 °C) can be prepared by the sol‐gel method,^[^ ^21^, ^25^ ^]^ indicating that commercial GDC cannot be used in their process. There are rare reports of sinter behavior and physicochemical mechanisms for multidoped ceria‐based materials, which limit the design and utilization of ceria‐based electrolytes for actual device fabrication. Additionally, most of the results of sintering behavior for doped GDC were prepared in an air atmosphere, as in previous studies.^[^ ^6^, ^27^, ^28^, ^29^, ^30^ ^]^ Considering the effect of oxidation at high temperatures for low‐cost steel base material, researching multiple doped ceria‐based materials sintered in an inert atmosphere is essential.

To reduce the high sintering temperature and enhance the ionic conductivity of GDC, the use of sintering aids and codopants has been widely investigated. Among them, Li‐ and Co‐based additives have shown great promise. Lithium‐containing compounds such as LiNO3 and Li2O have been reported to significantly lower the sintering temperature of GDC to below 1000 °C by forming transient liquid phases that enhance grain boundary mobility, even at doping levels around 2.5–5 mol%.^[^ ^20^, ^21^ ^]^ On the other hand, cobalt oxide (CoO) has been recognized as a highly effective sintering aid due to its volatility and its ability to promote densification without severely impacting phase stability.^[^ ^31^, ^32^ ^]^ Studies have shown that 1–5 mol% CoO doping not only reduces sintering temperature to 850–900 °C but also improves the electrical conductivity by increasing the grain boundary ionic transport.^[^ ^31^ ^]^ However, higher CoO content above 5 mol% may lead to conductivity degradation due to vacancy trapping and possible electronic conduction effects.^[^ ^32^ ^]^ Based on these findings, a codoping strategy using 5 mol% Li and 5 mol% Co was adopted in this work to explore the synergistic effects of both dopants at the high end of their effective doping range, while maintaining phase purity and achieving high performance at reduced processing temperatures.

In this study, for the first time, a lithium–cobalt dual‐doped GDC composite was synthesized in an inert atmosphere using commercial GDC powder as the starting material. The doping concentrations were set at 5 mol% lithium and 5 mol% cobalt, resulting in a material hereafter referred to as 5LC‐GDC. This composition was designed to simultaneously enhance sinterability and ionic conductivity while maintaining structural compatibility with conventional SOEC components. Remarkably, the 5LC‐GDC sample achieved a relative density of 95.53% at a sintering temperature as low as 650 °C, which, to the best of our knowledge, represents the lowest reported sintering temperature for doped GDC under conventional sintering conditions. The study also investigated the integration of 5LC‐GDC into a tubular metal‐supported cell, providing new insights into codoped ceria electrolytes that facilitate the fabrication of next‐generation, efficient solid oxide steam electrolyzers for sustainable hydrogen production. The fabrication method involved using conventional stainless steel porous tubes (grade 310S) to create tubular metal‐supported steam electrolyzers, which can remarkably reduce the device production costs.

As part of the analytical approach of this work, the effects of a high ratio (up to 10%) of Li and Co doping fractions in commercial GDC were investigated at various sintering temperatures. Thermogravimetric analysis (TG/DTA), Raman spectroscopy, field emission scanning electron microscopy (FESEM) with energy dispersive spectroscopy (EDX), and X‐ray diffraction (XRD) were employed to investigate the physicochemical characteristics. Thermodilatometry measurements were utilized to detect the sintering behaviors of calcinated powders, and the electrochemical performance of the sintered pellets was measured using electrochemical impedance spectroscopy (EIS) analysis. The result of this study could help better understand the electrochemical behavior of metal‐supported SOECs under dynamic operating conditions, enabling the design of the next generation of scalable steam electrolyzers from the design of functional materials to fabrication strategies.

## 2 Results and Discussion

**Figure 1:**
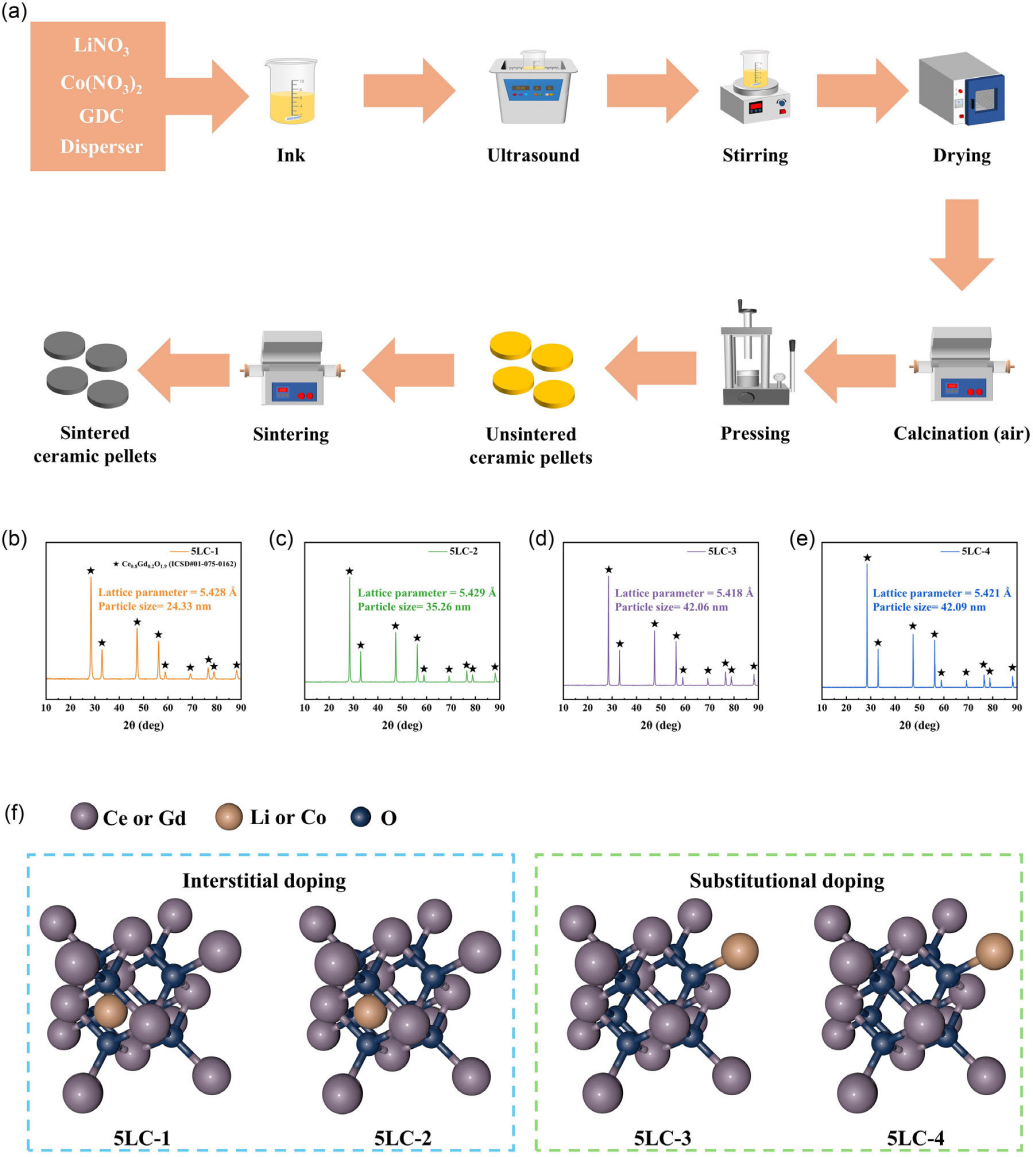
a) Fabrication process of the doped GDC pellets. b–e) XRD patterns of 5LC‐1, 5LC‐2, 5LC‐3, and 5LC‐4. f) Schematic diagram of doping types for 5LC samples with different sintering temperatures.

A synthesis method without ball‐milling was applied to prepare the doped GDC powder, as shown in **Figure** **1**a. Full experimental procedures, including synthesis parameters and materials specifications, are provided in detail in the Supporting Information. **Table** **1** shows the sintering conditions for ceramic pellets for 5LC‐GDC. In this study, the codoped GDC samples are denoted as 5LC‐1, 5LC‐2, 5LC‐3, and 5LC‐4, corresponding to sintering temperatures of 650 °C, 750 °C, 850 °C, and 950 °C for 6 h, respectively.

**Table 1:** Sintering conditions and the ratio (atom%) of the elements from the EDX analysis for 5LC pellets.

Sample	Conditions of sintering [°C]	Elemental ratio	O K	Co K	Ce L	Gd L
5LC‐1	650 (6h)	Atom %	39.05%	5.97%	41.81%	13.16%
		Atom % error	±0.7	±1.0	±0.6	0.8
5LC‐2	750 (6h)	Atom %	28.93%	4.07%	51.75%	15.25%
		Atom % error	±0.5	±0.7	±0.7	±1.0
5LC‐3	850 (6h)	Atom %	31.53%	4.37%	49.14%	14.96%
		Atom % error	±0.5	±0.6	±0.7	±1.0
5LC‐4	950 (6h)	Atom %	41.99%	3.10%	42.41%	12.50%
		Atom % error	±0.5	±0.5	±0.6	±0.8

### 2.1 Characterization of Thermogravimetry

The calcination temperature was set by the thermogravimetric analysis (TGA‐DSC) profiles. Figure S1a, Supporting Information, shows the TGA‐DSC profiles of GDC‐nitrate salt powder. The whole decomposition process of the mixture of GDC‐nitrate salts can be divided into three parts. The first part, starting from 20 °C up to 81.3 °C, could be due to the dehydration of the sample with loss of the adsorbed water and 2‐propanol trapped in the solid polycrystalline structure, with a weight loss of ≈5.31%. From the DSC curve, the first part of the process is an endothermic reaction. Ranging from 81.3 to 278.8 °C, it is the second part of the whole decomposition, which shows the largest weight loss (≈8.36%). The drop in weight is due to the preliminary decomposition of cobalt nitrate and PVP.^[^ ^33^, ^34^ ^]^ With the sintering temperature going above 278.8 °C, the decomposition process steps into the third part. In the third part, cobalt nitrate, lithium nitrate, and PVP undergo complete decomposition.^[^ ^29^, ^33^, ^35^, ^36^ ^]^ The weight loss stops at 586.09 °C, which indicates the organic components and nitrate salts have been almost fully decomposed. Therefore, based on the TGA‐DSC results, the calcination temperature is set at 600 °C in order to minimize the influence of the decomposition of nitrate salts and organic components.

### 2.2 Thermal Expansion Analysis

Figure S1b, Supporting Information, displays the linear shrinkage and the shrinkage rate of 5LC powder, which illustrates the sintering behavior of this material. The calcined powder started to shrink at ≈550 °C. The linear shrinkage curve of 5LC‐powder showed a single‐step densification behavior with the maximum shrinkage rate at 789.1 °C. Based on the curve, the shrinkage stops at 898.2 °C, which indicates the densification process of 5LC‐powder fully finishes at this temperature. As a reference, pure GDC powder starts to shrink at 1100 °C and only stops beyond 1350 °C.^[^ ^37^ ^]^ The results of thermal expansion analysis illustrate that the dual doping of lithium and cobalt obviously decreases the sintering temperature of GDC. In this material system, the doping of lithium can markedly enhance grain boundary mobility to increase the GDC's sinterability due to the large distortion of the surrounding lattice that facilitates defect migration.^[^ ^38^, ^39^ ^]^ The doping of Co can also increase the GDC's sinterability due to the melting of the dopant in the neck region of the particle contacts.^[^ ^12^ ^]^ Therefore, with the coordinating effect of lithium and cobalt on the sinterability of CGO, the 5LC powder obtained outstanding sinterability compared with pure GDC.

### 2.3 Structural Characteristics

The prepared ceramic pellets with different sintering temperatures (650, 750, 850, and 950 °C) are shown in Figure S1c, Supporting Information. As can be seen, the ceramic pellet shrinks with the temperature increase, consistent with the linear shrinkage results. The diameter of 5LC‐3 is close to that of 5LC‐4. This illustrates that the densification process of 5LC pellets nearly finishes at 850 °C.

The XRD results of 5LC pellets are demonstrated in Figure 1b–e. The crystal structure of pure GDC is calcium fluoride (CaF2), with the space group Fm3‐m (no. 225). The XRD result of pure GDC is obtained from the reference card (ICDD data, reference code: 01‐075‐0162). For the pure GDC, there are nine peaks in its XRD result, which present the presence of the (111), (200), (220), (311), (222), (400), (331), (420), and (422) planes of the face‐centered cubic (FCC) fluorite type structure. It is observed that all XRD results of 5LC pellets fit well onto GDC, which means there is only a single crystalline phase in the samples with a cubic fluorite structure, like GDC. No peaks of the other phases show the homogeneities of all 5LC samples, which are excellent, with no (or insignificant) impurities detected by XRD.

Based on Bragg's equation (Equation S1, Supporting Information), the doped GDC samples’ lattice constants are calculated, as displayed in Figure 1b–e. Compared with pure GDC (5.423 Å), all doped GDC samples showed distortions. It is commonly believed in previous studies for single Li‐doped GDC or Co‐doped GDC that the doping atom (Li or Co) usually substitutes Ce4+ or Gd3+ in the crystal structure, which leads to the lattice shrinkage effect.^[^ ^21^, ^31^ ^]^ It is because the ionic radiuses of Ce4+ (6‐coordinate, 1.01 Å) and Gd3+ (6‐coordinate, 1.078 Å) are larger than Li+ (6‐coordinate, 0.9 Å), Co2+ (6‐coordinate, 0.79 Å), and Co3+ (6‐coordinate, 0.69 Å). But here, in our research, we first find a different mechanism for the Li, Co doping in GDC. As can be seen, for the 5LC‐1 and 5LC‐2, the lattice parameters are 5.428 Å and 5.429 Å, larger than pure GDC (5.423 Å). This illustrates that the Li and Co ions come into the crystal lattice rather than substitute the positions of Ce or Gd ions with sintering temperatures of 650 and 750 °C. When the sintering temperature is up to 850 °C, the lattice parameters of 5LC‐3 reduce to 5.418 Å, which means the doping type of Li and Co ions has been changed from interstitial doping to substitutional doping. The lattice parameters of 5LC‐4 slightly increase to 5.421 Å, close to the pure GDC, possibly due to the partial evaporation of Li and Co at a relatively high sintering temperature (950 °C). Therefore, the doping type of Li, Co ions in GDC possibly could be inferred from the difference of lattice parameter, which is summarized in Figure 1f. The doping is interstitial with relatively low sintering temperature (650 and 750 °C); the interstitial doping will change to substitutional doping when the sintering temperature rises to 850 and 950 °C. These observations may suggest a possible transition from interstitial to substitutional doping as the sintering temperature increases, particularly given the reduction in lattice parameter at higher temperatures. However, this interpretation is based on indirect evidence from XRD data. Direct confirmation of the doping mechanism would require advanced structural characterization techniques such as EXAFS, EELS, or SAED, which were not available under the current experimental conditions.

The particle sizes of the samples are estimated by the Scherrer equation (Equation S2, Supporting Information), noted in Figure 1b–e. In previous studies, higher sintering temperatures would increase the particle size of GDC.^[^ ^24^, ^40^ ^]^ For the 5LC samples, the particle size is also increased by a higher sintering temperature. It can be seen that the particle size only shows a little increase from 5LC‐3 to 5LC‐4, which illustrates that the densification process of 5LC pellets nearly finishes at 850 °C, corresponding with the linear shrinkage results and diameter results of ceramic pellets.

### 2.4 Morphological Characteristics

**Figure 2:**
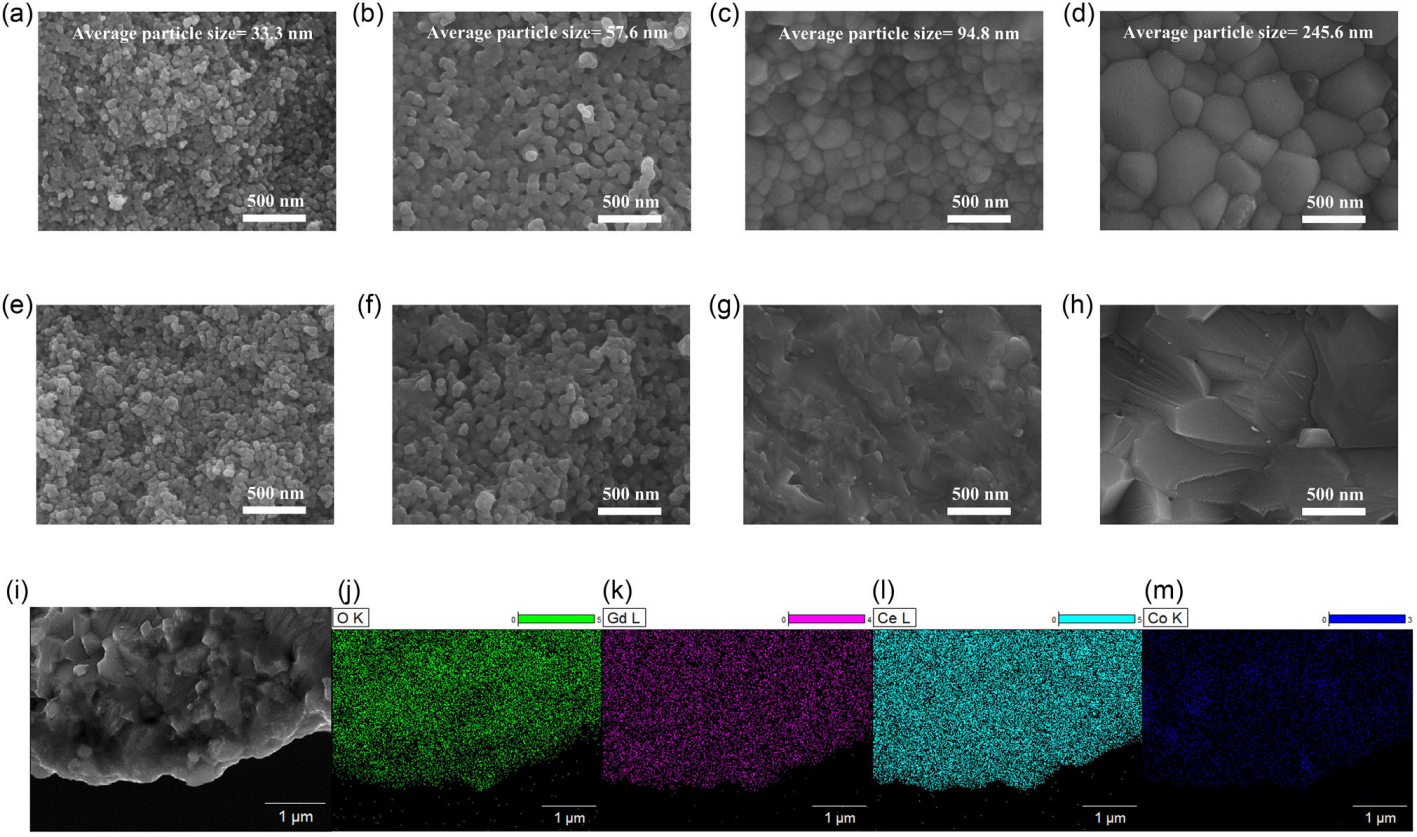
a–d) Surface SEM images of 5LC‐1, 5LC‐2, 5LC‐3, and 5LC‐4. e–h) Cross‐section SEM images of 5LC‐1, 5LC‐2, 5LC‐3, and 5LC‐4. i–m) EDX elemental mapping of the cross‐section for the 5LC‐4.

The surface morphologies of the 5LC pellets are shown in **Figure** **2**a–d with different sintering temperatures. In Figure 2a, all GDC grains have been connected after sintering at 650 °C, which means that the densification of GDC is promoted even at 650 °C by doping Li and Co. However, there are still some small pores between GDC grains, and the connection necks between GDC grains are relatively small. With the sintering temperature increasing to 750 °C, it is illustrated in Figure 2b that the connection necks between GDC grains become stronger, and many fewer pores can be found between GDC grains. In Figure 2c,d, all grains are connected well, and no pores or gaps can be found. According to particle size analysis (analyzed using the Nano measurer), the average sizes of particles for 5LC‐1, 5LC‐2, 5LC‐3, and 5LC‐4 are 33.3, 57.6, 94.8, and 245.6 nm. As the temperature during sintering rises, the average size of the particles on the sample surfaces also increases. The morphologies in the cross‐section of the 5LC pellets are shown in Figure 2e–h. With the sintering temperature at 650 and 750 °C, the morphologies in the cross‐section are similar to the surface morphologies. For the 5LC‐3 and 5LC‐4, a few grain boundaries and abundant broken crystal grains can be observed, which means the combination between GDC grains is strong enough to stay even when the crystal grains are cracked by external force. Energy‐dispersive spectroscopy (EDX) results excluding Li, Au, and C are shown in Table 1. The 5LC‐1 has the highest proportion of Co, and the proportion of Co stays at a similar value as the sintering temperature at 750 and 850 °C. The lowest proportion of Co is in 5LC‐4, possibly due to the slight evaporation of Co. EDX mapping results are presented in Figure 2i–m, which indicates the elemental distribution at the cross section of the 5LC‐4. From these results, the distribution of Co is relatively homogeneous in the GDC crystalline structure, cross‐confirmed by the XRD results. Also, a good homogeneity of the Gd element is observed in the electrolyte composite after the doping with Li and Co. No aggregations of Li, Co, or Gd are observed.

### 2.5 X‐Ray Photoelectron Spectroscopy

**Figure 3:**
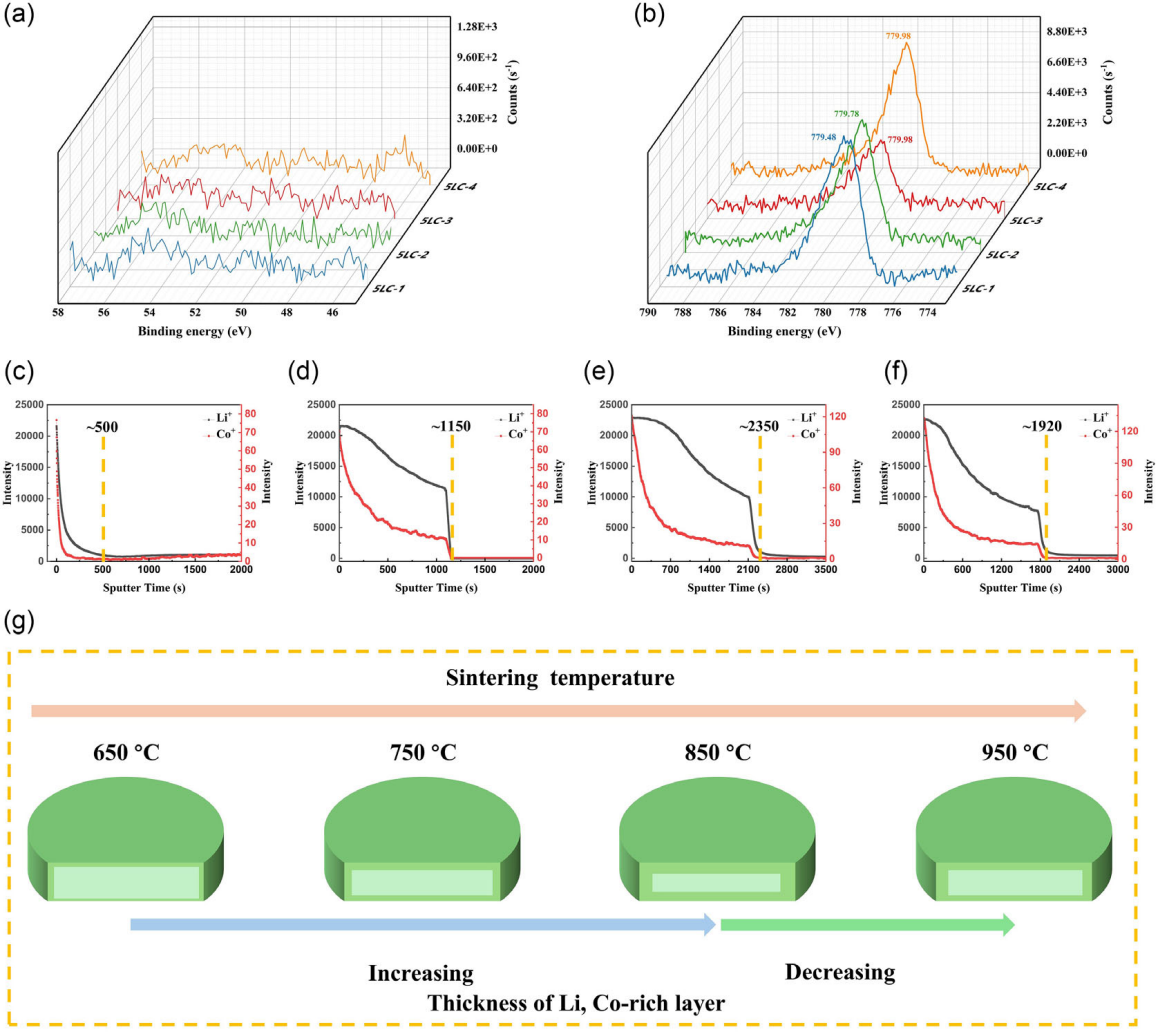
a) Li1s of XPS elemental spectra for 5LC samples. b) Co2p3/2 of XPS elemental spectra for 5LC samples. c–f) The ToF‐SIMS spectra for 5LC‐1, 5LC‐2, 5LC‐3, and 5LC‐4. g) The schematic diagram of distribution for Li, Co‐rich layer.

**Figure** **3**a shows the XPS elemental spectra position range in which the Li1s peaks should be located for 5LC samples. As can be seen, there are no observable Li 1s peaks in the XPS spectra. This could indicate either that the amount of Li present at the surface is below the detection limit of XPS or that lithium has partially evaporated or redistributed during the sintering process. Further analysis is required to distinguish between these possibilities. Co 2p3/2 is detected on the surface for all 5LC samples, as displayed in Figure 3b. The binding energy of Co 2p3/2 is ≈779.5 eV for the Co3O4^[^ ^41^ ^]^ and ≈780.0 eV for the Co2O3 or LiCoO2.^[^ ^42^, ^43^, ^44^, ^45^ ^]^ Considering the absence of Li peaks in the surface, the binding energy of Co 2p3/2 at ≈780.0 eV can only be the Co2O3. Therefore, based on the results of Co 2p3/2, the 5LC sample has Co3O4 on the surface with a sintering temperature of 650 °C. With the sintering temperature increasing to 750 °C, the binding energy of Co 2p3/2 for 5LC‐2 is at ≈778.0 °C, which indicates part of Co3O4 is converted into Co2O3. The binding energies of Co 2p3/2 for 5LC‐3 and 5LC‐4 are at ≈780.0 eV, which means there is only the Co2O3 on the surface.

### 2.6 Time‐of‐Flight Secondary Ion Mass Spectrometry

In order to further explore the depth distribution of doped elements on the surface and the existence of Li, the time‐of‐flight secondary ion mass spectrometry (ToF‐SIMS) is used, and the results are shown in Figure 3c–f. Compared with the EDX mapping, ToF‐SIMS has a much higher resolution for the depth distribution of elements, which can reach the nanometer level.^[^ ^46^ ^]^ Hence, the information on the depth distribution of doped elements on the surface can be illustrated by the ToF‐SIMS results.

ToF‐SIMS depth profiling shows strong Li^+^ signals at the outermost surface, even before sputtering, confirming that lithium is indeed present at the surface. This indicates that the absence of Li in the XPS spectra is more likely due to the limited sensitivity of XPS to lithium, rather than actual surface depletion.

Also, the amount of Li and Co continually decreases with the sputtering time and stabilizes later, which means there is a thin Li and Co‐rich layer for all 5LC samples. With the sintering temperature increasing from 650 to 850 °C, the thickness of the thin Li, Co‐rich layer continues to increase. The thickness of the layer decreases in the 5LC‐4, which means the sintering temperature (950 °C) of 5LC‐4 is high enough and evaporates part of the thin Li, Co‐rich layer. The schematic diagram of the distribution for Li, Co‐rich layer is intuitively summarized in Figure 3g.

### 2.7 Raman Spectra

**Figure 4:**
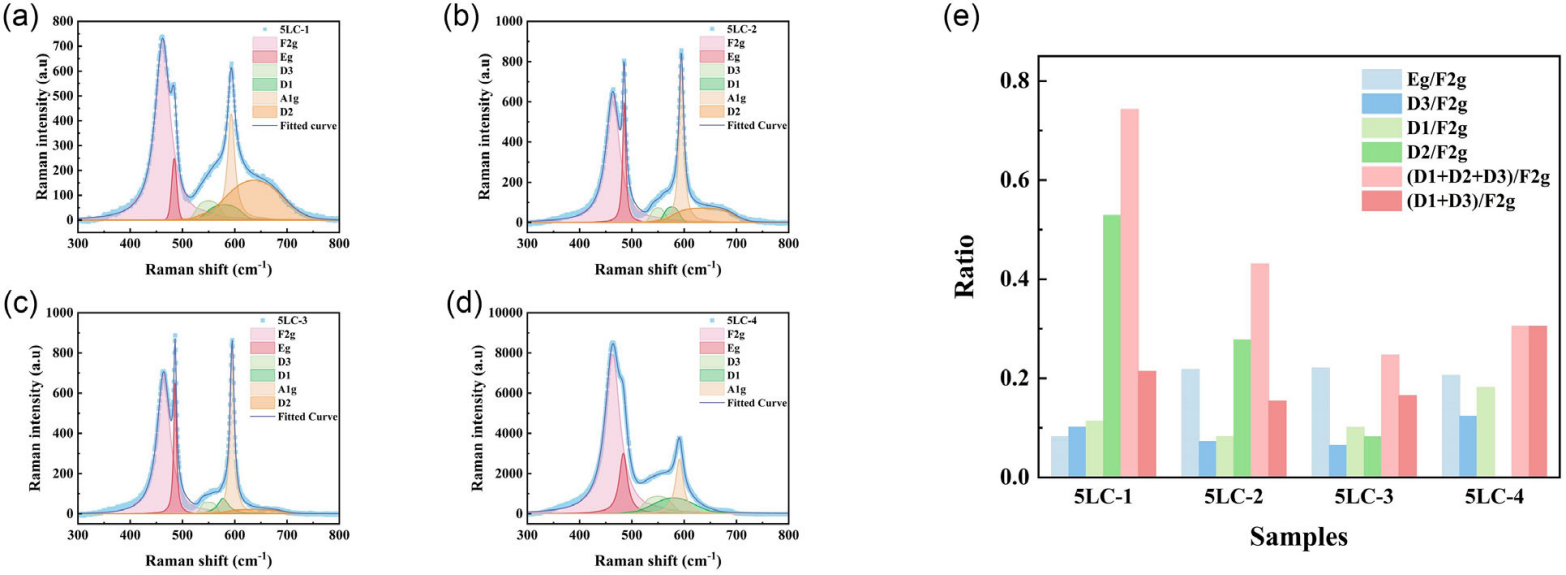
a–d) Raman spectra for 5LC‐1, 5LC‐2, 5LC‐3, and 5LC‐4. e) Area ratios (based on the area of F2g peak) of peaks from fitted Raman spectra for 5LC samples.

The impact of Li, Co doping on the GDC structure and the existence of new defective sites with different sintering temperatures is studied by Raman spectroscopy analysis, as shown in **Figure** **4**a–d. For the ceria‐based materials, there are four peaks that usually can be detected, which are “F2g,” “D1,” “D2,” and “D3,” respectively. The F2g peak is located at around 464 cm^−1^ and could always be detected in the Raman spectrum of ceria‐based material, which is ascribed to the symmetric stretch mode of the Ce‐O8 crystal unit.^[^ ^47^, ^48^, ^49^, ^50^, ^51^ ^]^ The D1 peak is located at 580–590 cm^−1^, related to the oxygen vacancies dealing with a Frenkel anion pair due to the moving of an oxygen atom into an octahedral interstitial position generating a vacancy.^[^ ^52^, ^53^, ^54^, ^55^ ^]^ The D1 peak can be considered an intrinsic defect in the structure of pure ceria.^[^ ^56^ ^]^ The peak center, at about 550 cm^−1^, is the D3 peak, which is usually assigned to the oxygen vacancies coupled with the presence of Ce3+ or other aliovalent cations.^[^ ^55^, ^56^, ^57^ ^]^ The D2 peak can be detected at ≈637 cm^−1^, which is linked to extrinsic defects created by the dopant's addition.^[^ ^55^, ^56^ ^]^ As can be seen, the D2 peak cannot be observed in Figure 4d, which means the extrinsic defects are reduced remarkably with the sintering temperature at 950 °C.

In Figure 4a–d, two other peaks are observed. The peaks at ≈485 and ≈595 cm^−1^ are named “Eg” and “A1g,” which are attributed to oxygen vibrations in LiCoO2 involving mainly O—Co—O bending (Eg) vibrations and Co—O stretching (A1g).^[^ ^58^ ^]^ It illustrates that LiCoO2 exists in all 5LC samples. Combined with the XRD results, it can be inferred that LiCoO2 may exist with an amorphous phase in the grain boundaries (or the amount of LiCoO2 is too low to be detected by XRD). Referring to the ToF‐SIMS and XPS results, for all 5LC samples, part of Li is doped into the crystal structure of GDC, and another Li exists in the LiCoO2. The distribution of Co is more complex. On the surface, Co exists in the cobalt oxide. In the interior, part Co is doped into the crystal structure of GDC, and another Co exists in the LiCoO2.

To further investigate the impact of sinter temperature on the 5LC samples, the Raman curves are fitted, and the area proportions of peaks (based on the area of the F2g peak) are displayed in Figure 4e. The area ratios of D1/F2g, D2/F2g, and D3/F2g can be used as the reference indexes for the amounts of D1, D2, and D3 defects in 5LC samples. The area ratios of (D1 + D2 + D3)/F2g are listed to show the total number of defects in 5LC samples caused by the doping of Li and Co with different sintering temperatures. The area ratio of Eg/F2g demonstrates the amount of LiCoO2 generated in the 5LC samples. The 5LC‐1 shows the lowest amount of generation for LiCoO2 and the highest ratio of D2/F2g, which suggests that the dopant's addition generates the most extrinsic defects. Figure 4e illustrates that 5LC‐3 shows the highest amount of LiCoO2. Notably, the intensity of LiCoO2‐related Raman peaks is higher in the 5LC‐3 sample than in 5LC‐4, indicating a higher amount of this phase. Given its absence in XRD patterns, this phase is likely nanocrystalline or amorphous. If present at grain boundaries, LiCoO2 may affect oxygen ion transport either positively or negatively, depending on its distribution and continuity. The impact of this phase on electrochemical performance will be discussed in the following sections.

As mentioned earlier, the D1 peak and D3 peak are related to the oxygen vacancy. Therefore, the area ratios of (D1 + D3)/F2g can reveal the amount of oxygen vacancy in the 5LC samples. Among 5LC samples, 5LC‐4 has the highest area ratios of (D1 + D3)/F2g, which can be inferred that 5LC‐4 has more oxygen vacancy compared with other samples. Hence, 5LC‐4 may show better ionic conductivity.

### 2.8 Relative Density

**Figure 5:**
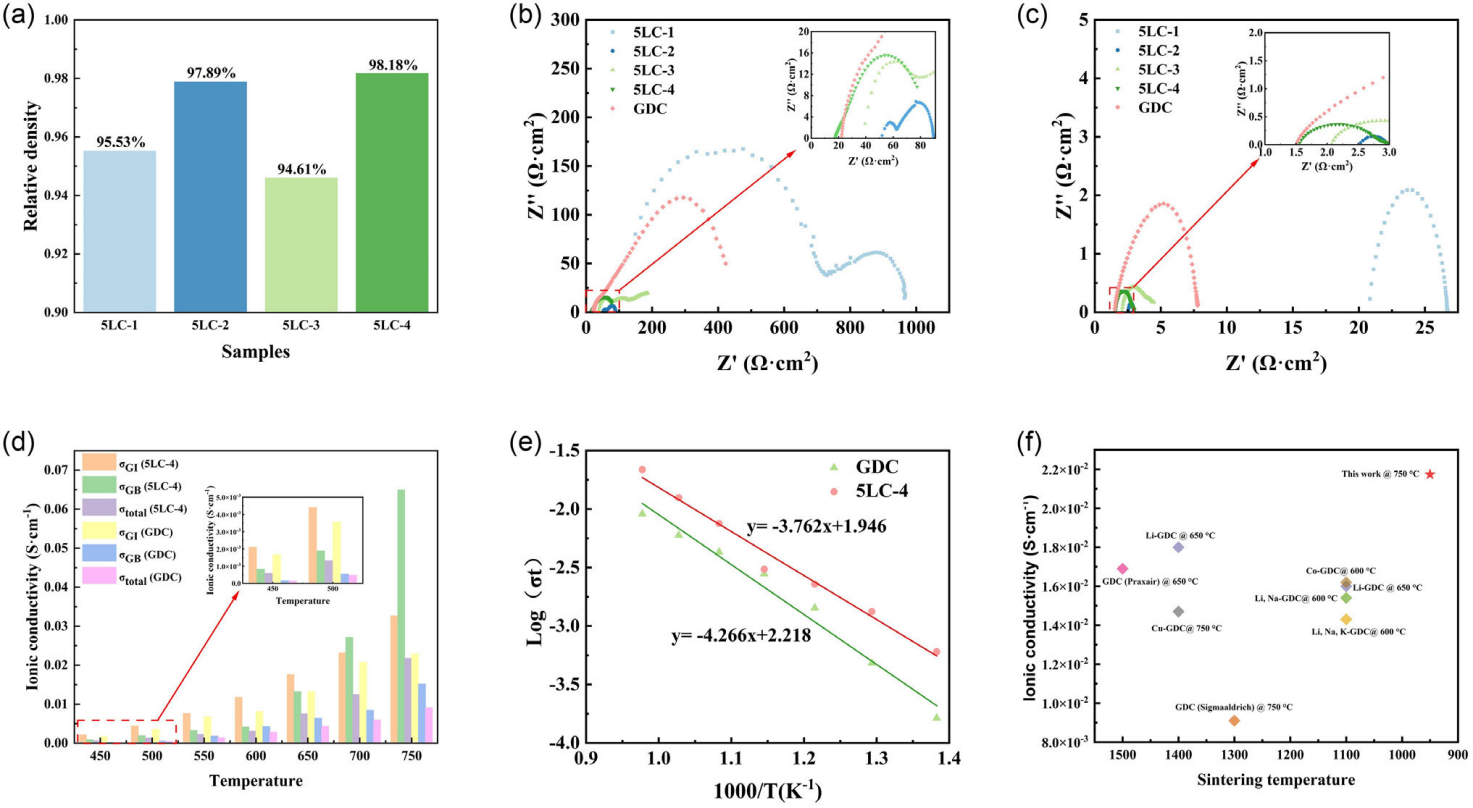
a) Relative densities of 5LC samples. b) Nyquist plots of 5LC samples at 450 °C (the inset shows the enlarged view of high‐frequency part). c) Nyquist plots of 5LC samples at 750 °C (the inset shows the enlarged view of high‐frequency part). d) Ionic conductivities at different temperatures for GDC and 5LC‐4. e) Arrhenius plot of the GDC and 5LC‐4. f) The comparison of total conductivity for different dopants between this work (5LC‐4) and previous studies.

The theoretical densities are estimated by the crystallographic equation (Equation S3, Supporting Information). The pycnometer is used to measure the densities of samples. Based on these data, the relative densities of 5LC samples are displayed in **Figure** **5**a. For the application of this ceria‐based material in SOECs, the sintered ceramic electrolyte layer is required to be gas‐tight enough to prevent gas diffusion between cathode and anode, which means the prepared ceramic pellets should be dense enough (relative density >95%) after sintering. As shown in Figure 5a, 5LC‐1 and 5LC‐2 show relative densities of ≈95.53% and 97.89% respectively, beyond 95%. However, as observed in the corresponding SEM images, some interconnected pores still remain, especially in the samples sintered at lower temperatures. If applied in a full cell, such residual porosity could potentially compromise ionic conductivity and reduce the open‐circuit voltage due to impaired gas‐tightness.

The relative density of 5LC‐3 is ≈94.61%, below 95%. However, according to the SEM results, the 5LC‐3 is firm enough to be used as the electrolyte layer in the SOECs. The relative density of 5LC‐3 should be larger than that of 5LC‐1 and 5LC‐2 because the SEM images of 5LC‐3 look dense and firm. This anomaly can be due to the generation of LiCoO2 and the calculation of the theoretical density. In the calculation, the Li and Co are assumed to be fully doped in the GDC, ignoring the generation of other phases. Therefore, the estimated theoretical densities should be larger than the “real” ones. Also, the amount of LiCoO2 is highest in the 5LC‐3, which means the value of its relative density is the most affected. Thus, from the relative density results, it can be found that 5LC‐3 and 5LC‐4 are dense enough as the electrolyte layer. 5LC‐1 and 5LC‐2 may be applied in the SOECs, but they face issues with the ionic conductivities and open circuit voltage.

### 2.9 Electrochemical Analysis of 5LC Pellets

The electrochemical characteristics (Nyquist plots) of 5LC samples and commercial GDC are illustrated in Figure 5b,c. The typical Nyquist plot of electrolyte material consists of three semicircles; each semicircle represents a distinct process, as displayed in Figure S3a, Supporting Information. The semicircles could be ascribed to the bulk of grains (high frequency), the grain boundaries (intermediate frequency), and the electrolyte‐current collector interfaces (low frequency).^[^ ^59^, ^60^ ^]^ The three semicircles may become one or two semicircles due to the relaxation behavior of the bulk and the grain boundaries, combined with the interference of experimental apparatus inductances.^[^ ^61^ ^]^ In visual observation, 5LC‐2, 5LC‐3, and 5LC‐4 show much lower ionic resistances compared with commercial GDC. This benefits from more oxygen vacancies in the GDC crystal structure created by the codoping of Li and Co. 5LC‐1 shows high ionic resistances among 5LC samples and GDC, which may be due to the poor connection between grains and the relatively low amount of oxygen vacancies. 5LC‐3 has relatively higher ionic resistances than 5LC‐2. From the Raman results, 5LC‐2 and 5LC‐3 have similar amounts of oxygen vacancies. One reason for the difference in ionic resistances for 5LC‐2 and 5LC‐3 is that 5LC‐3 has the thickest Li, Co‐rich layer, which increases the interface resistance. Interestingly, although Raman spectroscopy revealed a more pronounced LiCoO2 signal in the 5LC‐3 sample compared to 5LC‐4, the total ionic conductivity of 5LC‐3 was slightly lower. This indicates that the presence of LiCoO2 does not necessarily enhance ionic conduction. In fact, excessive LiCoO2, especially if distributed along grain boundaries, may hinder oxygen ion transport by partially blocking conduction pathways or increasing grain boundary resistance. These results suggest that the amount and distribution of LiCoO2 must be carefully optimized to avoid adverse effects on electrochemical performance.

It can be found that 5LC‐4 shows the lowest ionic resistance at the testing temperatures of 450 and 750 °C. The EIS result of 5LC‐4 confirms our previous speculation that 5LC‐4 should have a better ionic conductivity because of the highest amount of oxygen vacancies compared with other samples. The ionic resistances of all samples have decreased considerably with increasing operating temperature.

To further analyze the ionic conductivity, the Nyquist plots of 5LC‐4 and commercial GDC are chosen to fit with a typical equivalent circuit model. In the applied equivalent circuit, as shown in Figure S3a, Supporting Information, three subcircuits are in series, each containing a resistor (R) and constant phase elements (CPE) connected in parallel. Among the equivalent circuits, L1 represents the apparent inductor; RGI is the resistance of the grain bulk; RGB is the resistance of the grain boundary; Rin is the interface resistance of the electrolyte‐current collector. The fitted curves of 5LC‐4 and GDC are shown in Figure S2a–d, Supporting Information. Based on Equation S4, Supporting Information, the ionic conductivities of GDC and 5LC‐4 are calculated, as shown in Figure 5d. As can be seen, 5LC‐4 shows outstanding ionic conductivities compared with commercial GDC. The maximum total ionic conductivity obtained for the 5LC‐4 sample is 2.17 × 10^−2^ at 750 °C. With the testing temperature of 450 °C for 5LC‐4, the ionic conductivity of grain, grain boundary's ionic conductivity, and total ionic conductivity are 25.6%, 366.6%, and 269.5% larger than commercial GDC, respectively. At 750 °C, the ionic conductivity of grain, the ionic conductivity of grain boundary, and total ionic conductivity for 5LC‐4 are 42.9%, 329.5%, and 138.85% larger than commercial GDC. It shows that Li and Co doping obviously enhances GDC's ionic conductivity. The promotion of the ionic conductivity of the grain boundary is much larger than the ionic conductivity of the grain. This promotion is possibly because more Li and Co exist in the grain boundary, leading to more defects and oxygen vacancies. Also, the number of defects in the grain boundary is higher than in the grain interior, making it easier to capture the atoms of Li and Co into the structure.

Using the values of ionic conductivities and the Arrhenius equation (Equation S5, Supporting Information), the Arrhenius plots of 5LC‐4 and GDC are calculated, as displayed in Figure 5e, where the straight lines are the least squares fits of the total conductivity data with the linearized form of the Arrhenius equation. It can be found that the slope of the fit line for 5LC‐4 is larger than that of commercial GDC, which illustrates that 5LC‐4 has a lower activation energy than commercial GDC. Based on slopes and the Arrhenius equation (Equation S5, Supporting Information), the specific value of activation energy can be calculated. The activation energy for 5LC‐4 is 0.32 eV. The activation energy of commercial GDC (sintered at 1300 °C) is 0.37 eV. The activation energy of GDC would increase to 0.83 eV if using the same sintering temperature (950 °C) with 5LC‐4.^[^ ^38^ ^]^ The results illustrate that the 5LC‐4 has a low activation energy, which is a considerable improvement compared with GDC.

The comparison of total conductivity for different dopants between this work and previous studies is presented in Figure 5f.^[^ ^21^, ^24^, ^27^ ^]^ Various studies have been conducted to improve the conductivity of GDC by doping with alkali and transition metal elements. These dopants enhance ionic conductivity by increasing oxygen vacancy concentration. Among them, Li and Co dopants have shown relatively greater improvement in GDC conductivity. However, most of these studies report sintering temperatures exceeding 1000 °C, which remains too high for metal‐supported applications. In contrast, our current work (5LC‐4) demonstrates good conductivity at a sintering temperature below 1000 °C, indicating strong potential for application in metal‐supported solid oxide cells. Based on prior research, it is also evident that the supplier and synthesis method of GDC or doped GDC significantly influence the measured conductivity. For instance, the commercial GDC powder from Sigma–Aldrich exhibits only half the ionic conductivity compared to that from Praxair despite being fully sintered at 1300 °C. Therefore, it alone cannot be used as the sole criterion for evaluating the effects of doping. A more objective indicator would be the relative improvement in conductivity compared to the undoped sample from the same supplier or synthesized using the same method. From this perspective, the 5LC samples exhibit a significant increase in conductivity compared to the commercial GDC powder from Sigma–Aldrich, demonstrating the positive impact of Li and Co codoping.

### 2.10 Electrochemical Analysis of 5LC‐Cell

**Figure 6:**
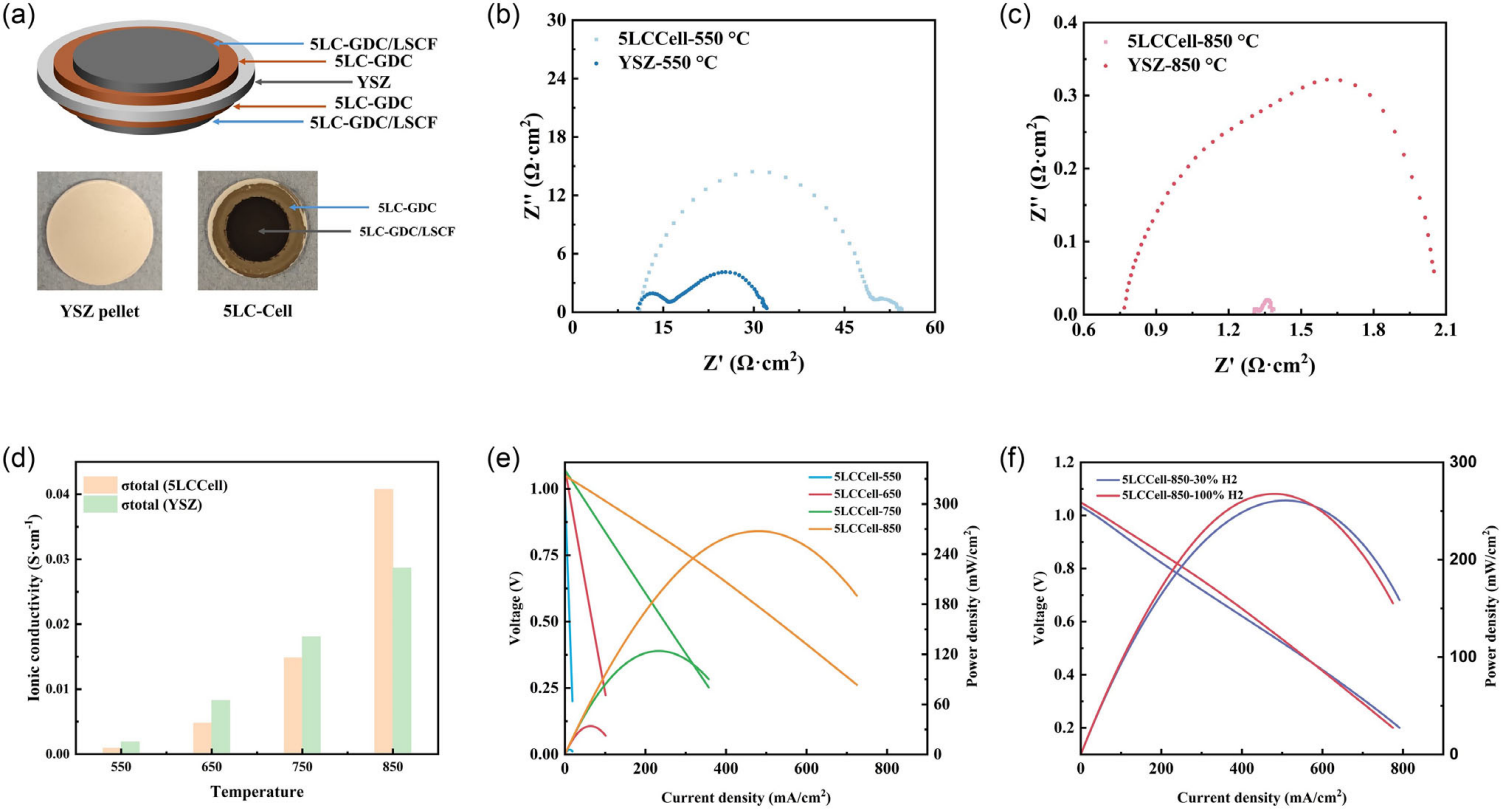
a) The schematic diagram of the prepared symmetric 5LC‐Cell, along with images of the YSZ pellet and the assembled 5LC‐Cell. b) Nyquist plots of 5LC‐Cell and YSZ pellet at 550 °C. c) Nyquist plots of 5LC‐Cell and YSZ pellet at 850 °C. d) Ionic conductivities at different temperatures for 5LC‐Cell and YSZ pellet. e) Potential and power density versus current density of 5LC‐Cell under 100% wet H2 (100 mL min^−1^) with different temperatures. f) Potential and power density versus current density of 5LC‐Cell under 30% and 100% wet H2 (100 mL min^−1^) at 850 °C.

To evaluate the performance of 5LC‐doped GDC in a solid oxide cell, a sandwich‐type symmetric cell was fabricated using a YSZ pellet (as shown in **Figure** **6**a) as the electrolyte support. In this configuration, the YSZ support is an electronic insulator to prevent potential electronic conductivity from the 5LC‐GDC layer. Figure 6a presents a schematic of the symmetric 5LC‐cell comprising five layers. The sintering temperature for both the 5LC‐GDC layers and the 5LC‐GDC/LSCF composite layers is set at 950 °C, based on the previous EIS results of 5LC pellets. After sintering, the 5LC‐GDC layers appeared brown, while the 5LC‐GDC/LSCF layers turned black. EIS measurements are conducted on both the 5LC‐cell and the YSZ support over a temperature range of 550–850 °C. Figure 6b shows the impedance spectra at 550 °C, where the YSZ support exhibits lower impedance than the 5LC‐cell. However, as shown in Figure 6c, a significant reduction in the impedance of the 5LC‐cell occurs at 850 °C, resulting in a lower impedance than that of the YSZ support.

The significant reduction may be attributed to diffusion between the 5LC‐GDC layers and the YSZ support. Previous studies have widely reported this diffusion phenomenon.^[^ ^62^, ^63^, ^64^ ^]^ Generally, interdiffusion between GDC and YSZ leads to the formation of solid solutions with lower ionic conductivity than that of pure GDC or YSZ, thereby reducing the overall conductivity and performance of the cell.^[^ ^65^ ^]^ However, in this study, the diffusion between 5LC‐GDC and YSZ appears to positively affect the bulk cell performance. This improvement is likely due to the high doping levels of Li and Co, which enhance the ionic conductivity of the solid solution formed at the 5LC‐GDC|YSZ interface at 850 °C.

To further analyze the total ionic conductivity, the Nyquist plots of the 5LC‐Cell and YSZ are fitted using equivalent circuit models. The YSZ data are modeled using the same circuit equivalent to the 5LC samples (Figure S3a, Supporting Information). For the 5LC‐Cell, a typical equivalent circuit model of the whole cell (Figure S3b, Supporting Information) featuring two distinct semicircles is used, corresponding to two sets of (R//CPE) elements connected in series. In this model, Ro represents the ohmic resistance of the whole cell, RCT is the charge‐transfer resistance, and RID denotes the interfacial and diffusion resistance. The fitted curves are shown in Figure S3c–f, Supporting Information. Figure 6d illustrates the total ionic conductivities at different temperatures for the 5LC‐Cell and YSZ pellets. It can be seen that the conductivities of the two are close at 750 °C, and at 850 °C, the total conductivity of the 5LC‐Cell has exceeded that of the YSZ pellet. These results suggest that the 5LC‐GDC layer acts as a promoter for YSZ, significantly enhancing the total ionic conductivity.

The *I*–*V* and *I*–*P* curves in SOFC mode are displayed in Figure 6e with different testing temperatures. The open‐circuit voltages (OCVs) stabilize at ≈1.01–1.07 V for 5LC‐Cell when temperatures increase from 550 °C to 850 °C, and the maximum power density is 267.5 mW cm^−2^ @ 0.556 V at 850 °C. Figure 6f shows the *I*–*V* and *I*–*P* curves with different gas inputs at 850 °C. The nearly identical curves under 30% and 100% wet H_2_ (100 mL min^−1^) at 850 °C suggest that the electrode layers and the LSCF catalyst have reached a performance bottleneck, likely due to the relatively large particle size of LSCF (20–500 μm). As only conventional ball milling is employed for ink preparation, particle size reduction to the nanoscale is not achieved. Therefore, such performance values are expected, as the cell fabrication is not optimized for performance testing. For example, the electrolyte is relatively thick (≈620 μm), and the electrode layers have fewer reactive sites.

### 2.11 Exploration of Fabrication for Tubular Metal Support Cells and Discussion

To further investigate the application of 5LC‐GDC on metal supports, the fabrication of tubular metal‐supported cells is carried out. Porous 310SS tubular metal substrates (Figure S4a, Supporting Information) serve as the supporting structure. Compared to the fabrication of planar electrolyte‐supported cells, the development of tubular metal‐supported cells presents greater challenges. Cracking and delamination of the coated layers after sintering represent the primary issues. As shown in Figure S4b, Supporting Information, these failures initiate in the 5LC‐GDC/NiO electrode layer after sintering. The problem becomes more severe when the 5LC‐GDC electrolyte layer is applied, leading to significant cracking and delamination and, in some cases, complete detachment of the small parts of layers from the metal support, as illustrated in Figure S4c, Supporting Information. At first, the issue is attributed to the electrode ink formulation. Therefore, various ink compositions are tested, as summarized in Table S1, Supporting Information. The ink formulation for the 5LC‐GDC electrolyte remains the same as that used in the 5LC‐Cell. However, the electrode ink formulation adjustments do not alleviate the cracking and delamination problem. Another problem is the weak combination between the metal support and the electrode layer. The part of the layers without delamination can be easily wiped down by blue tissues with IPA, as illustrated in Figure S4d, Supporting Information.

To investigate the causes of cracking, delamination, and poor interfacial adhesion between layers, XRD analysis is conducted on the metal support, as shown in Figure S5a, Supporting Information. The diffraction peaks match well with the standard XRD pattern of austenite (Figure S5b, Supporting Information), indicating that austenite is the primary phase in the metal support. Due to its porous structure, the XRD pattern exhibits a high background noise level. If present in small quantities, minor phases cannot be identified through XRD alone. Therefore, complementary characterization techniques, including SEM and EDX, are employed to provide further insights.

Figure S6, Supporting Information, presents the surface characterization of the metallic support. Figure S6a, Supporting Information, shows a low‐magnification SEM image, revealing the overall surface morphology and porous support structure. A higher magnification image is provided in Figure S6b, Supporting Information, highlighting the finer surface features and microstructural details. SEM analysis reveals the presence of numerous spherical particles distributed on the 310 SS skeletal structure. From the EDX results (Figure S6c–h, Supporting Information), the numerous spherical particles consist of Si and C. This common segregation phenomenon in steel occurs when the silicon and carbon contents are too high or when the cooling rate is too rapid.^[^ ^66^, ^67^ ^]^

The presence of Si‐ and C‐containing phases on the as‐received 310SS substrate prior to any coating is a key factor influencing interfacial adhesion. These segregated phases originate from the alloy composition and manufacturing process of the stainless steel, where silicon and carbon can precipitate at grain boundaries or on pore surfaces during solidification and cooling. Several intrinsic characteristics of these phases contribute to weak bonding at the metal/ceramic interface during low‐temperature sintering. First, Si and C phases possess very high melting points (Si ≈ 1414 °C; SiC > 2000 °C), which prevents them from softening or reacting with the applied electrode/electrolyte layer under the relatively low sintering temperatures (<1000 °C) used in this work. Second, Si/C phases often form smooth, glassy, or carbide‐like surfaces with low surface energy, which reduces the wetting and adhesion of screen‐printed or slurry‐cast inks at room temperature prior to sintering. Third, the chemical and structural stability of these phases at intermediate temperatures limits interdiffusion and chemical bonding across the interface, thereby preventing the development of strong metallurgical or ionic bridges between the coating and the metallic support. These combined effects make it challenging to achieve robust adhesion of the functional layers to the 310SS substrate at low sintering temperatures. Consequently, controlling or removing Si/C‐rich phases will be an essential step for improving the interfacial strength of tubular metal‐supported cells. To achieve this, feasible strategies include chemical surface pretreatments (e.g., acid etching), the introduction of barrier or wetting interlayers, and the selection of alternative low‐Si stainless steel substrates with better ceramic compatibility.

Based on the combined analysis of the experimental results and shrinkage tests, the primary causes of cracking, delamination, and poor interfacial adhesion between the layers can be attributed to the following factors: 1) Si and C phases significantly reduce interfacial adhesion between the functional layers and the metallic support. During the sintering process, it is challenging to establish a strong bond between the Si/C phases and the electrode layer due to the high melting points of silicon (1414 °C) and silicon carbide (>2000 °C), which prevent effective diffusion or chemical bonding. 2) The 5LC‐GDC layer exhibits a relatively high shrinkage rate (≈17%), which induces considerable internal stress during sintering. If the interfacial adhesion between the functional layers and the metallic support is insufficient, this stress can lead to cracking and delamination. Notably, the 5LC‐GDC electrolyte layer exhibits more severe cracking and delamination than the electrode layer, primarily due to the higher internal stress generated during its sintering process.

The fabrication strategy would be advantageous following a comprehensive characteristics and optimization analysis in a separate follow‐up study in the future. Therefore, as future work, the cell fabrication will be optimized, and its performance under various operating conditions will be measured. The primary optimization points identified are the shrinkage rate and surface composition, both of which are crucial factors influencing the quality of immaculate tubular metal‐supported cells.

## 3 Conclusions

This study investigated the synthesis, characteristics, and application of a high‐level (5% each) Li and Co codoped GDC for the fabrication of metal‐supported solid oxide steam electrolyzers. Structural, microstructural, and electrochemical analyses showed uniform dopant distribution without aggregation. XRD confirmed successful incorporation of Li and Co into the GDC lattice, influenced by sintering temperature, with no secondary phase formation. However, the proposed transition in doping mechanism from interstitial to substitutional is based on lattice structural trends and remains a hypothesis; further validation using advanced characterization techniques such as EXAFS or EELS is planned in future studies.

Thermal and microstructural investigations demonstrated that Li and Co codoping significantly reduced the sintering temperature of GDC. Dense microstructures were observed at sintering temperatures of 850 °C and 950 °C. ToF‐SIMS indicated a Li, Co‐rich surface layer whose thickness depended on the sintering temperature. High relative densities of 95.53% and 98.18% were achieved at 650 °C and 950 °C, respectively.

Raman spectroscopy identified LiCoO_2_ phases and linked oxygen vacancy concentration to sintering conditions. Although the formation of a LiCoO_2_ surface phase was not confirmed by XRD due to its potentially low crystallinity or limited quantity, Raman spectroscopy and ToF‐SIMS analyses provided consistent evidence of a Li–Co‐rich surface layer. These findings suggest the possible formation of an amorphous or nanocrystalline LiCoO_2_ phase. While direct microstructural verification via TEM could not be conducted under the current experimental conditions, further high‐resolution characterization is planned in future work to confirm the phase identity and distribution. Electrochemical impedance spectroscopy showed enhanced ionic conductivity with optimized sintering temperatures. The sample sintered at 950 °C (5LC‐4) had the highest ionic conductivity, outperforming commercial GDC by 269.5% at 450 °C and 138.85% at 750 °C. The maximum conductivity of 5LC‐4 reached 2.17 × 10^−2^ S cm^−1^ at 750 °C, with an activation energy of 0.32 eV. The 5LC‐GDC layer improved the ionic conductivity and performance of YSZ support, particularly at 850 °C. The cell achieved a peak power density of 267.5 mW cm^−2^ and stable OCVs, highlighting the potential of 5LC‐GDC in advanced SOFCs. The study also examined 5LC‐GDC in tubular metal‐supported SOECs, identifying shrinkage behavior and interfacial composition as crucial parameters. These findings suggest that high‐ratio Li and Co codoped GDC is a promising electrolyte material for metal‐supported SOECs, offering high conductivity and low sintering temperatures, which will aid in designing next‐generation SOECs. Exploring the effects of different Li and Co codoping ratios may be a valuable direction for future research to further optimize the electrolyte's performance. Additionally, in‐depth structural characterization techniques such as high‐temperature XRD, EXAFS, EELS, and SAED could provide further insight into phase evolution and dopant incorporation mechanisms. Finally, long‐term stability testing—including extended EIS measurements and redox cycling under operating conditions—will be carried out in future work to comprehensively evaluate the durability and practical applicability of 5LC‐GDC electrolytes in solid oxide electrolysis cells.

## Supporting Information

The authors have cited additional references within the Supporting Information.^[^ ^68^ ^]^

## Conflict of Interest

The authors declare no conflict of interest.

## Supporting information

Supplementary Material

## Data Availability

The data that support the findings of this study are available in the supplementary material of this article.
