# Supplementary material for: Li—Co Dual‐Doped Ceria‐Based Composite as a Promising Low‐Temperature Electrolyte for Metal‐Supported Solid Oxide Electrolyzers
Source: ChemSusChem. 2025 Nov 3;19(2):e202501679. doi: 10.1002/cssc.202501679 (PMC12854247; doi:10.1002/cssc.202501679)
Supplement: Supplementary file 1 — Supplementary Material [file CSSC-19-e202501679-s001.pdf]

Li-Co Dual-Doped $Ce_{0.8}Gd_{0.2}O_{1.9}$ Composite as a Promising Low-Temperature Electrolyte for Metal-Supported Solid Oxide Electrolysers

Supporting Information

Yuheng Liu[a], Ming Xu[b], Wei Zhang[b], Yunlong Zhao[c], Bahman Amini Horri[*a]

Experimental Section

Reagents

GDC powder (nanopowder, contains 20 mol % gadolinium as dopant), lithium nitrate (LiNO3, ≥99.99%), cobalt nitrate hexahydrate (Co (NO3)2 · 6 H2O, ≥98%), polyvinylpyrrolidone (PVP, average mol wt 40,000), 2-propanol (analytical grade, ≥99.5%), zirconium(IV) oxide-yttria stabilised (YSZ, nanopowder, contains 8 mol % yttria as stabiliser), α-Terpineol (90%, technical grade), ethyl cellulose (EC,46 cP), lanthanum strontium cobalt ferrite (LSCF, 20-500 μm, 6428), carbon (spherical powder, 2-12 μm, ≥99.95%) were bought from Sigma Aldrich (Merck) and used as precursors without further purification. Stainless steel tubular porous metal supports (grade 310S), featuring 10 μm open porosity and dimensions of 120 mm in length, 8.5 mm outer diameter, and 4 mm inner diameter with 1/8 NPT threading at both ends, were supplied from Carbis Filtration Ltd, UK. The ethylene glycol, polyethylene glycol (PEG) and polyvinyl butyral (PVB) also were bought from Sigma Aldrich (Merck).

Synthesis method

A synthesis method without ball-milling was applied to prepare the doped GDC powder, as shown in **Error! Reference source not found. (a)**. Drying, calcination, pressing, and sintering were the preparation processes used to make ceramic powders and ceramic pellets.

Preparation of mixed ceramic powder

3g mixture of lithium nitrate (5 mol%), cobalt nitrate hexahydrate (5 mol%), GDC (90 mol%) were mixed with 7mL 2-propanol. 0.3g PVP is added as the dispenser. The mixture became a stable, homogeneous ink via 10 min of ultrasound followed by 24 h of stirring at room temperature. The ink was dried at 80 °C in the oven to get homogeneous GDC-nitrate salt powder.

Preparation of calcinated ceramic powder

The GDC-nitrate salts powder was calcinated in the muffle furnace in air at 600 °C for 2 hours to remove the carbon and organic chemicals in order to obtain the Li, Co-doped GDC powder (5LC-powder). This composition is referred to as 5LC-GDC throughout the study, indicating 5 mol% Li and 5 mol% Co co-doped GDC.

Preparation of ceramic pellets

The calcined electrolyte powders were compressed using a manual hydraulic press (2-10 MPa pressure) with uniaxial dies (∅=3-15 mm) to create several disc-shaped green bodies with a 0.3-0.5 mm thickness. To prepare the ceramic pellets, these green bodies were sintered under flowing argon gas in a tube furnace. **Error! Reference source not found.** shows the sintering conditions for ceramic pellets. Also, the commercial GDC powder was used to fabricate ceramic pellets as a reference for electrochemical characterisations, with a sintering temperature of 1350 °C (6h).

The YSZ pellets were prepared in a similar way, with a sintering temperature of 1400 °C (6h).

Preparation of electrolyte ink and electrode ink

A 10 mL solution was prepared using ethyl cellulose (EC) and α-terpineol, followed by continuous stirring for 24 hours to ensure complete dissolution of EC. Subsequently, 5LC-powder was added to the solution, and the resulting mixture is subjected to ball milling for 72 hours to produce the electrolyte ink. The electrode ink was prepared similarly, using a 50:50 volume ratio of 5LC powder and LSCF, with the addition of 0.2 g of carbon per 10 mL of the mixture.

Preparation of symmetric 5LC-Cell

The electrolyte ink was applied to both sides of the YSZ pellet using a brush-coating method, followed by drying at 125 °C. This coating and drying process was repeated three times for each side. Subsequently, the coated YSZ pellet is sintered at 950 °C for 6 hours to form the 5LC-GDC electrolyte layers. The electrode layer was prepared using a similar procedure, with a final sintering step at 950 °C for 2 hours.

Characterisation

Physicochemical characterisation

The crystallite results of the samples were detected using an X-ray powder diffractometer (XRD, PANalytical X'Pert3; Cu-Kα radiation, λ = 1.5406 Å, 40 kV and 30 mA) in the range 10° ≤2θ ≤ 90° with a scanning rate of 1.3° min−1. The morphologies of the samples were investigated via a field-emission scanning electron microscope (FESEM, JEOL 7100F; 15 kV, 8 A) equipped with an EDX detector (Oxford Instruments). For the FESEM, the samples were coated with a 10 nm layer of gold to get clear images. TGA-DSC was tested using a TA Instruments SDT-Q600 instrument (room temperature to 1000 °C at 5 °C min−1 in atmospheric air). The

prepared samples were detected by Raman spectroscopy in the range of 200–3000 cm−1 via a Thermo DXR2 spectrometer (excitation wavelength 532 nm, 8 mm optical objective 50×). A push-rod vertical dilatometer (NETZSCH, DIL 402C) was also applied to test the linear shrinkage behaviour (room temperature to 1000°C, with a heating rate of 2 °C min−1 in atmospheric air). A gas pycnometer (AccuPyc II – 1345, Micromeritics) was utilised to measure the apparent densities of the sintered ceramic pellets. The methods for the calculation of lattice parameters, particle sizes and theoretical densities are shown in the SI. Time-of-Flight Secondary Ion Mass Spectrometry (ToF-SIMS) analyses were carried out on an ION-TOF GmbH (Münster, Germany) TOF.SIMS 5 instrument. The instrument was equipped with a reflectron-type analyser and microchannel plate detector. A bi-liquid metal ion source (LMIS) was employed to acquire mass data. Mass data is acquired using the Bi3+ cluster ion. XPS analyses were performed on a ThermoFisher Scientific (East Grinstead, UK) K-Alpha+ spectrometer. XPS spectra were acquired using a monochromated Al Kα X-ray source (hν = 1486.6 eV).

Electrochemical characterisation

The electrochemical impedance spectroscopy (EIS) of ceramic pellets was characterised by a potentiostat-galvanostat electrochemical workstation (Interface 1010E, Gamry, USA) in the frequency range from 0.1 Hz to 2 MHz at an AC voltage amplitude of 10 mV, and at temperatures ranging from 450 °C to 750 °C in an air atmosphere. The methods for the calculation of ionic conductivities and activation energy are shown in the SI.

Cell performance was evaluated using linear sweep voltammetry (LSV) with a Gamry system, recording polarisation curves from open-circuit voltage (OCV) to 0.2 V at a scan rate of 10 mV/s within a temperature range of 550–850 °C. The anode was connected to a silver wire, and the cell was hermetically sealed onto a custom-designed test fixture using silver paste. The cathode side was exposed to ambient air, while the anode side was supplied with either 30% humidified hydrogen (3 vol% H_2O) in argon or fully humidified hydrogen, both at a flow rate of 100 sccm. The active area of a single cell was approximately 0.21 cm².

Characterisation calculation details

Physicochemical characterisation

The lattice parameters are calculated from the following Bragg's equation[1]:

$$\alpha = d\left(\sqrt{h^2 + k^2 + l^2}\right) \quad \text{Equation S1}$$

$\boldsymbol{\alpha}$: cubic lattice parameter; $\boldsymbol{d}$:lattice spacing between the planes in the atomic lattice; $\boldsymbol{h}$, $\boldsymbol{k}$ and $\boldsymbol{l}$: the miller indices of the plane of diffraction.

The Scherrer's equation is employed to estimate the size of crystallites[1]:

$$D = \frac{0.9\lambda}{\beta cos\,(\theta)}$$ Equation S2

$\boldsymbol{D}$: crystallite size (nm); **λ**: X-ray wavelength (nm); **β**: line broadening at half maximum intensity (FWHM) (rads); **θ**: Bragg angle (rads).

Theoretical densities (ρ) are calculated from the following crystallographic equation:

$$\rho = \frac{\sum_i v_i * Z_i * M_i}{a^3 * N_A}$$ Equation S3

$\boldsymbol{v_i}$: stoichiometric coefficient; $\boldsymbol{Z_i}$: the number of atom $\boldsymbol{i}$ per unit cell (face-centred cubic for a ceria system); $\boldsymbol{M_i}$: the molar mass of atom $\boldsymbol{i}$ in g·mol−1; $\boldsymbol{a}$: the lattice parameter from XRD patterns;$\boldsymbol{N_A}$: Avogadro's number.

Electrochemical characterisation

The following equation is used to calculate the bulk conductivity (σ GI), grain-boundary conductivity (σ GB), and total conductivity (σ t) of the GDC samples:

$$\sigma = L/RA$$ Equation S4

$\boldsymbol{\sigma}$:the electrical conductivity; $\boldsymbol{L}$: the thickness of the prepared GDC pellet; $\boldsymbol{A}$: the electrode surface area contacting with the ceramic pellet (the silver coating); $\boldsymbol{R}$ (RGI, RGB, and Rt): resistances calculated by modelling analogous circuits.

The electrical conductivity and activation energy are estimated by a temperature-dependent Arrhenius conductivity model as below:

$$\sigma_T = \sigma_0\, e^{-Ea/kT}$$ Equation S5

$\boldsymbol{Ea}$: activation energy; $\boldsymbol{\sigma_T}$: the temperature-dependent electrical conductivity; $\boldsymbol{T}$: the absolute temperature; $\boldsymbol{K}$: the Boltzmann constant (1.36 ×10-23 J/K); $\boldsymbol{\sigma_0}$: a pre-exponential factor representing the intrinsic conductivity of the electrolyte polycrystalline structure.

Results and discussion

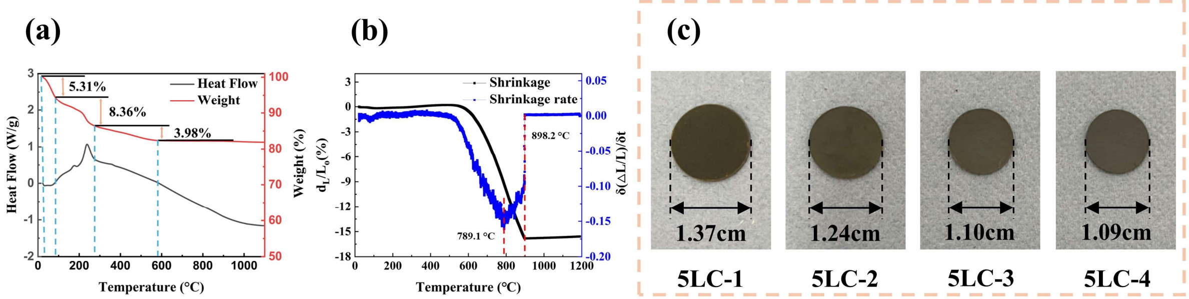

Figure S1. (a) TGA-DSC profiles of GDC-nitrate salts powder without calcination, (b) The linear shrinkage and the shrinkage rate of 5LC-powder, (c) Prepared ceramic pellets,

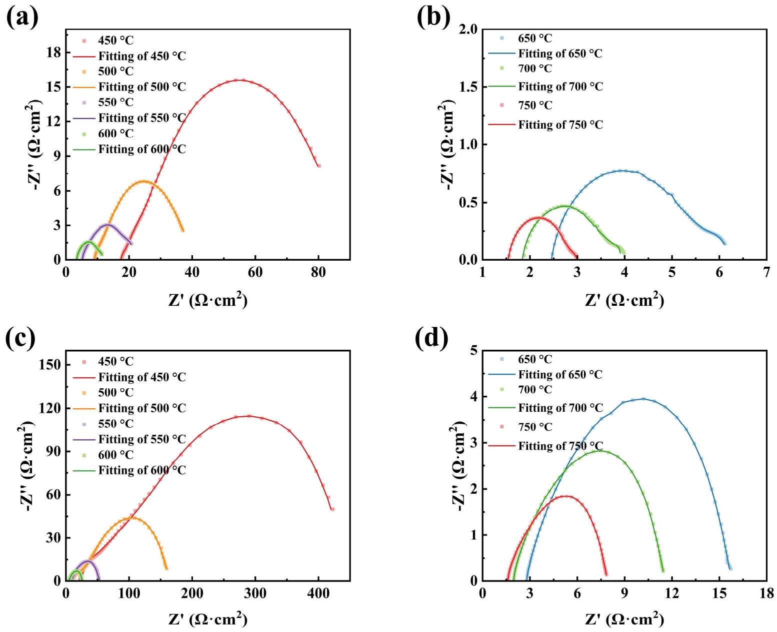

Figure S2. (a)Nyquist plots and fitting curves of 5LC-4 at 450, 500, 550, 600 °C, (b) Nyquist plots and fitting curves of 5LC-4 at 650, 700, 750 °C, (c) Nyquist plots and fitting curves of GDC at 450, 500, 550, 600 °C,(d) Nyquist plots and fitting curves of GDC at 650, 700, 750 °C.

WILEY-VCH

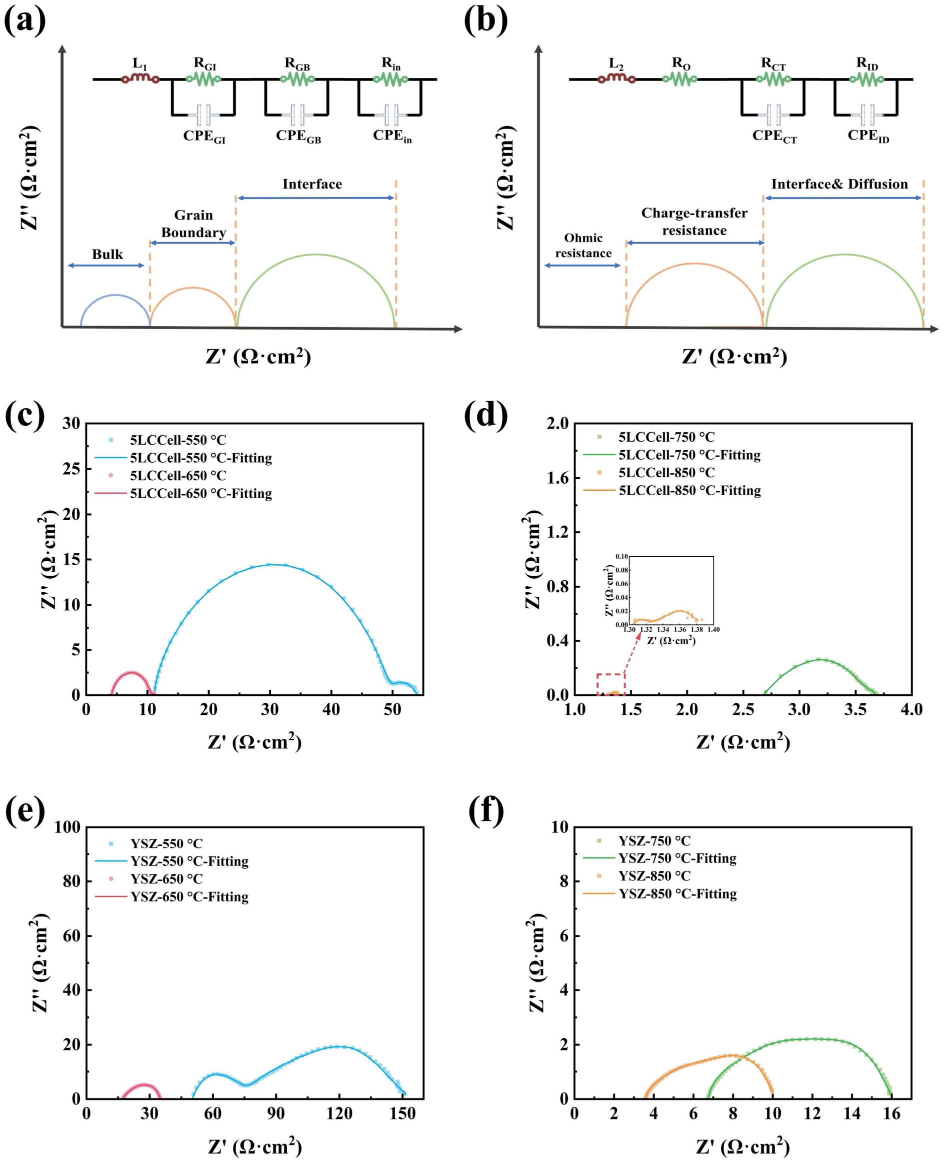

Figure S3. (a) Illustration of a typical impedance plot for a polycrystalline sample with equivalent circuits, (b) Illustration of a typical impedance plot for a symmetric cell with equivalent circuits, (c) Nyquist plots and fitting curves of 5LC-Cell at 550, 650 °C, (d) Nyquist plots and fitting curves of 5LC-Cell at 750, 850 °C, (e) Nyquist plots and fitting curves of YSZ at 550, 650 °C, (f) Nyquist plots and fitting curves of YSZ at 750, 850 °C.

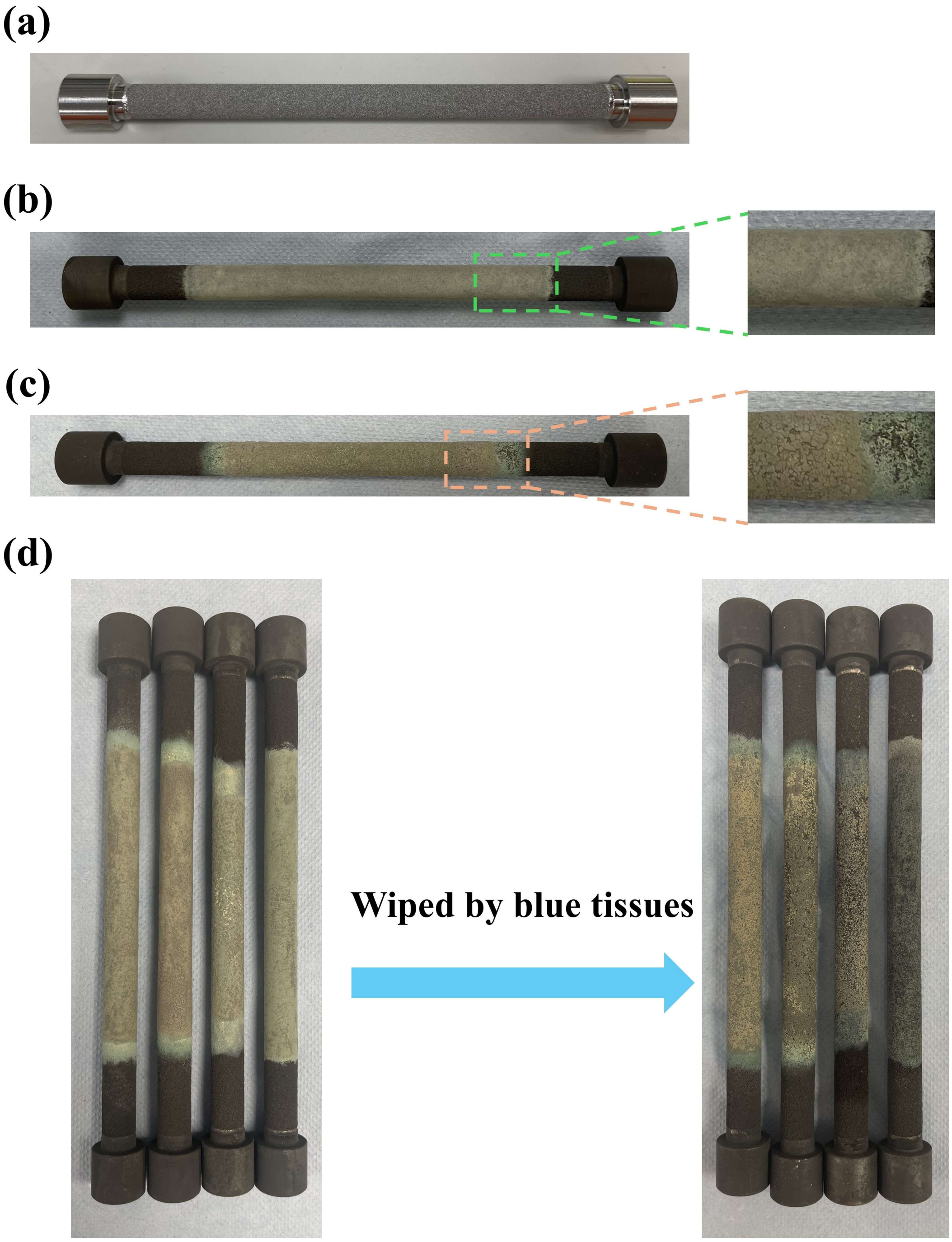

Figure S4. (a) Stainless steel (grade 310S) porous tubes, with 10 μm open porosity, 120 mm in length, 8.5 mm outer diameter, and 4 mm inner diameter with 1/8 NPT threading at both ends, as the SOEC cell support (b) a representative photo of the tubular metal support coated with a hydrogen electrode, (c) a representative photo of the tubular metal support coated with both the hydrogen electrode and the 5LC-GDC electrolyte, (d) a comparison photo showing the coated tubular metal support before and after when being wiped by blue tissue with IPA.

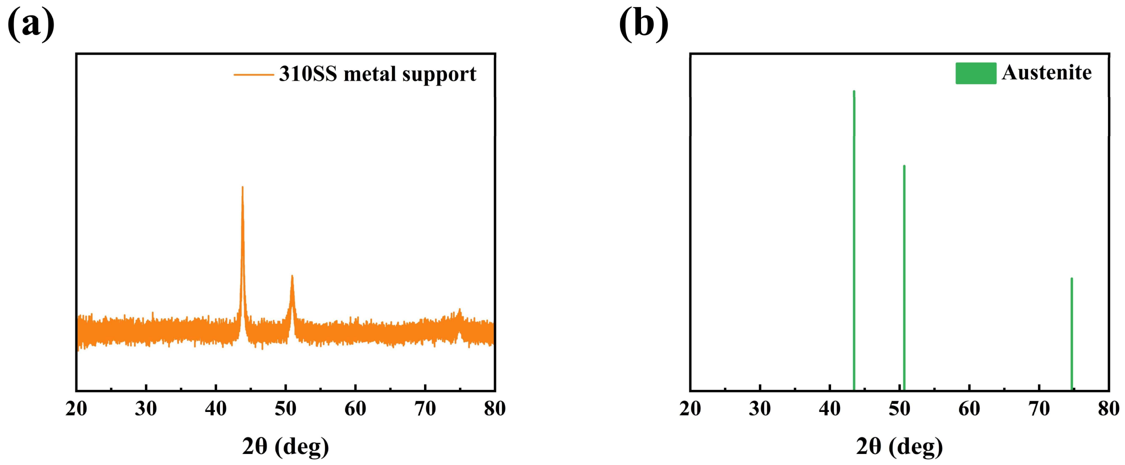

Figure S5. (a) XRD pattern of metal support. (b) standard XRD pattern of austenite (Reference code:00-023-0298)

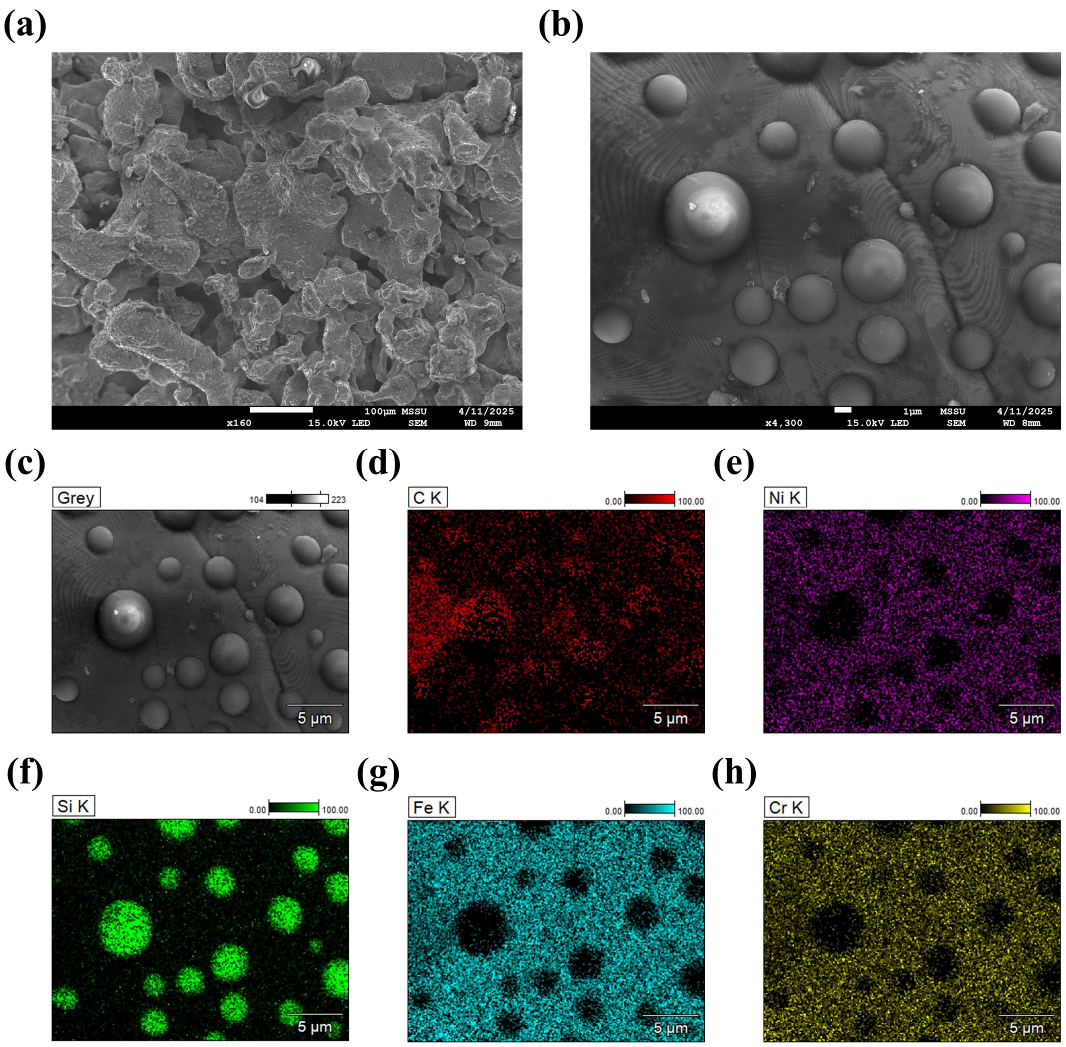

Figure S6. (a) Low-magnification SEM image of the metal support surface; (b) High-magnification SEM image of the metal support surface; (e–h) EDX elemental mapping of the metal support surface.

Table S1. Ink formulas for the NiO/5LC-GDC electrode

Material	Solvents	Dispenser	Binder	Plasticizer	Atmosphere	Sintering process	Results
5LC-GDC/NiO 50 v%: 50 v%	IPA 7mL H2O 2mL	PEG 0.2g	Ethyl cellulose (EC) 0.2g	a-Terpineol 1 to 5 mL Ethylene glycol 1 to 5 mL	Air and Ar both tried	950 °C 6h	Combination of powder is Weak.
5LC-GDC/NiO 75 m%: 25 m%	IPA 7mL	PVP 0.2g	Ethyl cellulose (EC) 0.2g	a-Terpineol 1 to 5 mL Ethylene glycol 1 to 5 mL	Air and Ar both tried	950 °C 6h	Cracked after sintering
5LC-GDC/NiO 75 m%: 25 m%	IPA 7mL H2O 2mL	PVP 0.2g	Ethyl cellulose (EC) 0.2g	a-Terpineol 1 to 5 mL Ethylene glycol 1 to 5 mL	Air and Ar both tried	950 °C 6h	Cracked after sintering
5LC-GDC/NiO 75 m%: 25 m%	IPA 7mL H2O 2mL	PEG 0.2g	Ethyl cellulose (EC) 0.2g	a-Terpineol 1 to 5 mL Ethylene glycol 1 to 5 mL	Air and Ar both tried	950 °C 6h	Cracked after sintering
5LC-GDC/NiO 75 m%: 25 m%	a-Terpineol 7 mL	-	-	-	Air and Ar both tried	950 °C 6h	Cracked after sintering
5LC-GDC/NiO 75 m%: 25 m%	a-Terpineol 7 mL IPA 2mL	-	-	-	Air and Ar both tried	950 °C 6h	Cracked after sintering
5LC-GDC/NiO 75 m%: 25 m%	a-Terpineol 7 mL IPA 2mL	-	-	-	Air and Ar both tried	950 °C 6h	Cracked after sintering
5LC-GDC/NiO 80 m%: 20 m%	a-Terpineol 7 mL IPA 2mL	-	PVB	-	Air and Ar both tried	950 °C 6h	Cracked after sintering

References

[1] I. Unal, S. Meisuria, M. Choolaei, T. R. Reina, B. A. Horri, *Ceramics International* **2018**, *44*, 6851-6860.

WILEY-VCH
